# A new model for standardising and treating thermal injury in the rat

**DOI:** 10.1016/j.mex.2019.09.006

**Published:** 2019-09-12

**Authors:** Lisa Davenport, Geoffrey Dobson, Hayley Letson

**Affiliations:** Heart, Trauma & Sepsis Research Laboratory, College of Medicine and Dentistry, James Cook University, Townsville, Queensland, 4811, Australia

**Keywords:** Severe thermal injury in the rat for evaluation of acute resuscitation, Burns, Rat model, Thermal injury, Prehospital

## Abstract

Thermal burn injury methodologies are inconsistently described within the current literature. To permit the advancement of new treatments there is an urgent need for the development and standardisation of an acute rat model. We describe a rat thermal burn model that involves: anaesthesia, chronic catheterisation, skin preparation, baseline hemodynamic and physiological monitoring, and a quantifiable method to reproduce a severe full-thickness burns injury affecting ∼30% percent of the total body surface area (%TBSA). Following a 15 min post-burn period, treatment commences with an acute monitoring phase lasting up to 8 h, which can be modified according to individual protocols. This model reflects the clinical continuum-of-care from point-of-injury, a 15 min ambulance response time, a 60 min prehospital phase and hospital treatment monitoring phase. The model is validated with histological evidence of full-thickness injury, evidence of the hypermetabolic response (K+, Base Excess, lactate) and changes in complete blood counts.

• It has been 50 years since Walker and Mason published their widely popular “A Standard Animal Burn Model”.
• The model, however, lacks quantifiable methodology for the assessment of burn thickness, surface area burnt and physiological status.
• We present a new standardised method for evaluation of drug and interventional therapies that mimic the clinical scenario including ambulance response, pre-hospital and hospital phases after burn.

## Graphical abstract

**Figure:**
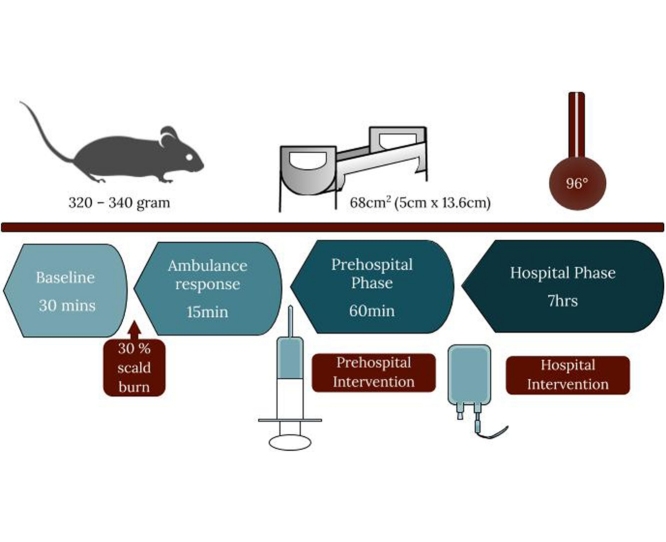

**Specification Table**

**Table:** 

Subject Area:	Medicine and Dentistry
More specific subject area:	Laboratory Preclinical
Method name:	Severe Thermal Injury in the Rat for Evaluation of Acute Resuscitation
Name and reference of original method:	Walker HL, Mason AD (1968) A standard animal burn. *J Trauma* 8(6):1049-51
Resource availability:	NA

## Method details

### Methodology background

The goal of resuscitation in the acute phase after severe burns is to preserve organ function while avoiding complications of ‘fluid creep’. The over resuscitation that leads to fluid creep and the associated complications such as compartment syndrome and pulmonary oedema has not been combated by any current treatment [1]. Existing research into improving the formulas and fluid type have only resulted in minor advancement since the original Parkland formula for fluid resuscitation was developed over 50 years ago [[2], [3], [4]]. A standardised preclinical severe burns model is needed to allow for new novel therapies and resuscitation protocols to be accurately evaluated.

Burn methodology used in preclinical studies often lacks detail that prohibits easy replication. A systematic review evaluating 116 studies concluded that most studies were poorly described and lacked reproducibility [5]. The standardisation of the severe burn will allow for better comparison between studies. A key consideration in standardising a burns model is the accurate calculation of the percentage of body surface area injured as it directly correlates with the degree of metabolic changes demonstrated on the blood gas and full blood count [6]. A full thickness burn terminates the sensation nerve terminals, reducing pain and discomfort associated with the burns wound, an important consideration for animal welfare. However, current full thickness burn studies widely vary on the reported exposure temperature and time; from 10 s cutaneous contact with 95 °C water [7] to 96 °C for 25 s [8]. Longer exposures result in greater damage to underlying structures below the dermal layers, making the comparison of organ damage difficult between studies. Standardising the thermal technique and exposure time will ensure that the desired burns thickness is achieved without directly impacting visceral organs.

Burn injuries occur in the community, delaying the time before an intervention can be received. To replicate this environment, preclinical studies of burn injury must, therefore, consider the delay in providing an intervention. The first responders’ arrival time varies around the world and is influenced by geographical isolation. Countries with a developed ambulance service report a response time of less than 15 min [[9], [10], [11]]. Time from ambulance response to hospital admission is also an important consideration when trying to replicate a real-world scenario. A standardised method should also incorporate the widely accepted first aid of severe burns, including cooling the burn for 20 min and covering the wound, which are standard around the world [12]. A severe burns model that reflects the clinical environment improves the translational potential of preclinical discoveries.

### Animal welfare declaration

All procedures were reviewed and approved by the James Cook University Animal Ethics Committee (Approval Number: A2479).

### Animal preparation and surgical instrumentation

10 Sprague-Dawley adult male rats (320–340 g) were obtained from James Cook University’s Breeding Colony, Townsville, Australia. All animals were housed in a 14 h–10 h light–dark cycle with free access to food and water ad libitum. Animals were anaesthetised with 5% isoflurane in oxygen via nose cone and were breathing spontaneously. To maintain anaesthesia continuous 0.5–1.5% isoflurane in oxygen was delivered, while monitoring for the absence of the withdrawal reflex and change in heart rate. Animals received 0.05 mg/kg Buprenorphine Hydrochloride (Temgesic Injection) subcutaneously between the shoulder blades (above the burn area) for pain relief.

Under anaesthesia, the hair was removed on the dorsal and lateral surfaces including the left inguinal area with clippers and Veet depilatory cream. The shaved area was disinfected with povidone-iodine solution and cleaned with 70% ethyl alcohol. All animals were placed on a homeothermic blanket (Physitemp, ADInstruments, Bella Vista, New South Wales, Australia) and core body temperature was monitored and recorded throughout the duration of the experiment using a rectal probe (T-type Pod, ADInstruments) to maintain a core temperature of 35.5–37.5 °C.

Sterile chronic catheters (Access Technologies, Skokie, IL, USA) were inserted in the left femoral artery and vein and attached to an Instech dual access vascular port fitted with a jacket (Walker Scientific, Perth, Western Australia). Venous access allowed for fluid infusion and arterial access allowed for blood sampling and blood pressure monitoring. To fit the chronic catheters a 12 mm transverse incision was made on the dorsal surface of the neck above the level of the shoulder blades, allowing for the creation of a subcutaneous pocket the length of the back to the level of the hips via blunt dissection. A further incision at the left inguinal area was made to allow for the blunt dissection in the subcutaneous space with long straight forceps, permitting placement of the catheters. Once inserted and secure the catheters were flushed with heparinised saline to ensure patency before closing the neck and inguinal incisions with 4-0 braided silk suture. This technique for femoral vascular access has previously been described [[13], [14], [15]]. The arterial catheter is connected to a pressure transducer and Bridge Amplifier/PowerLab (AD Instruments) for continuous blood pressure monitoring. Subcutaneous electrocardiogram (ECG) leads were also attached to the fore- and hind-limb paws for heart rate monitoring. After instrumentation, the animals remained anaesthetised for a 30-min stabilisation period prior to recording their baseline measurements.

### Full thickness burn injury

#### Surface area calculation

The total body surface area (TBSA) and total surface area burnt was calculated using the Meeh formula: TBSA = kW^2/3^, where k = 9.83, and W = weight in kg [16].

#### Thermal injury apparatus

**Fig. 1:**
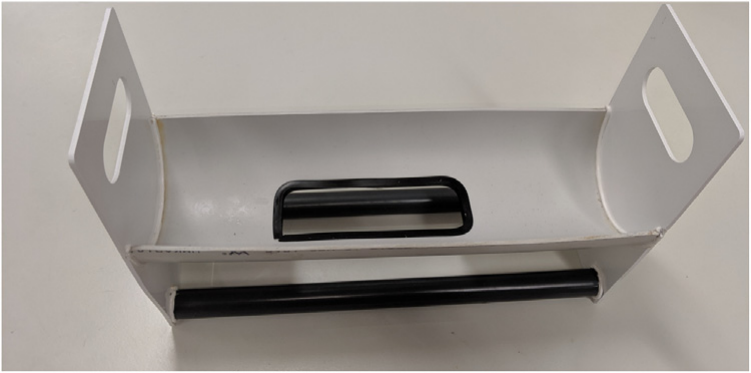
Custom Cradle made from Poly Viny Chloride (PVC) 100 mm pipe with a 3 mm wall thickness.

**Fig. 2:**
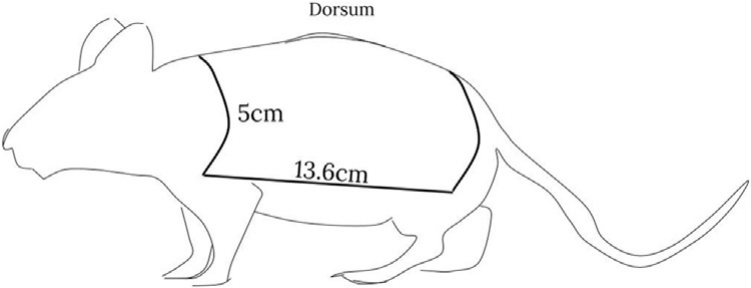
Left lateral surface with schematic representation of surface area.

**Fig. 3:**
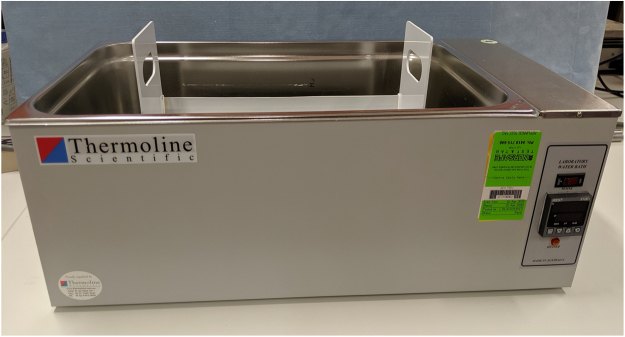
Cradle and Water Bath.

A cradle with a prefabricated aperture of 68 cm^2^ (5 cm × 13.6 cm) was created based on the selected rat weight range of 320–340 g (Fig. 1). The cradle was constructed from commonly available Poly Vinyl Chloride (PVC) 100 mm pipe with a 3 mm wall thickness to allow rubber seal to be secured around the aperture. The animal is placed in the cradle left lateral dorsal side down first followed by the right lateral dorsal side to achieve 28.4–29.6% full thickness burn (Fig. 2). The hold device fits in a scaffold, which ensures the device, and therefore the animal can only be lowered to just below the surface of the water bath and no deeper, for maximum protection (Fig. 3). The water bath (Thermoline Scientific Laboratory) temperature is set to 96 °C.

After baseline recording, the anaesthetised animal is disconnected from the Bridge AMP and ECG leads are temporarily removed. This allows the animal to be placed in the cradle with the left lateral dorsal side down while the isoflurane nose cone remains attached. Immediately prior to performing the thermal injury, two consecutive toe-pinch tests on two different limbs are performed to confirm the appropriate depth of anaesthesia. The cradle is then immersed into 96 °C water for 8 s, recorded on an electronic timer. Immediately after, the right lateral dorsal side is then exposed for 8 s, avoiding the paws, tail and head.

Following burn induction, the anaesthetised animal is immediately lifted out of the cradle and the dorsal surface dried gently with clean paper towel. The animal is then placed prone on a clean, dry sterile underpad over a homeothermic blanket set to 37 °C to maintain normothermia. Continuous hemodynamic monitoring is reconnected including arterial blood pressure, ECG, and rectal temperature probe.

#### Ambulance response phase

A simulation of the time for an ambulance to respond is observed for 15 min with no intervention. Only monitoring occurs, and animals remain anaesthetised on a maintenance dose of isoflurane (0.5–1.5%).

#### Prehospital phase

At the commencement of the 60-min prehospital phase, the anaesthetised animals have water applied for 20 min to the burned area, as per current standard-of-care treatment for burns patients. To simulate continuous running water a room temperature water spray bottle is used continuously for 20 min, while the animal is prone on the homeostatic blanket to maintain normothermia. The burn site is then covered with a 3 M Tegaderm Film (Medshop, Australia). At this time, it would be clinically appropriate to administer an intervention or resuscitation through the venous access to simulate a paramedic or first responder treating the burn injury prior to hospital admission.

#### Hospital phase

At the commencement of the hospital phase, the isoflurane was ceased, and the rectal probe and ECG leads were removed from the animal. The conscious animals in the recovery cage had access to water, softened food pellets, and DietGel Recovery (Clear H_2_O). A homeothermic blanket was placed under the cage to maintain warmth (37 °C) for the entire monitoring period. During the 7 -h hospital phase, animals were secured to the Instech Vascular Access Harness, swivel and tether system, allowing for continuous arterial monitoring. The temperature was recorded hourly using a non-invasive Infrared Digital Thermometer. During this phase, an intervention or fluid resuscitation therapy may be administered through the venous access to simulate medical therapy received during a hospital stay. The chronic catheters can remain in place for up to 40 days and the hospital phase can be shortened or extended dependent on individual study requirements.

#### Method validation

**Fig. 4:**
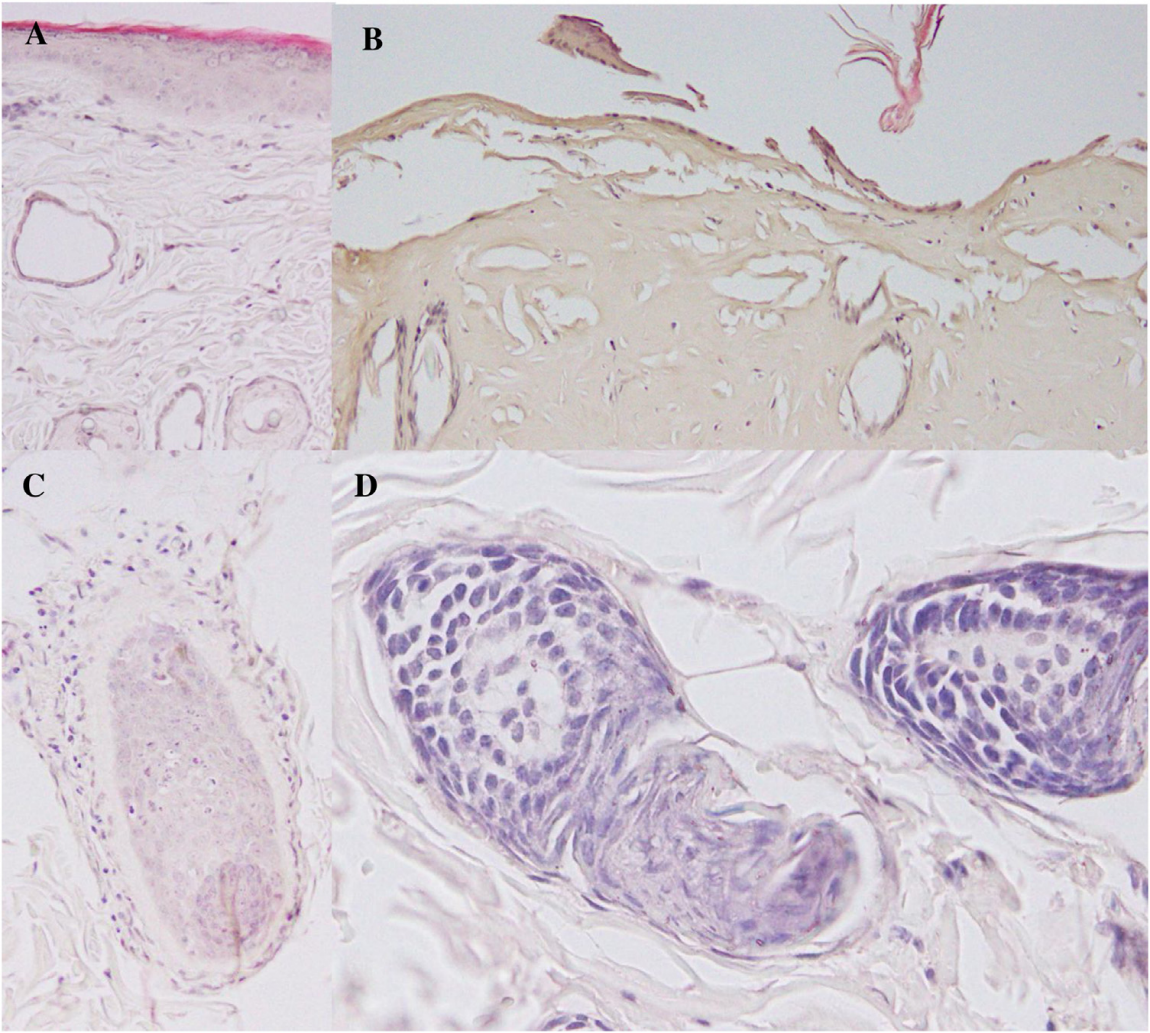
Deep thickness burn injury histology in Sprague-Dawley rats 8 h and 15 min after injury: A) Uninjured epidermis and dermis, 40 × . B) Burnt epidermis and dermis, 40 × . C) Uninjured hair follicle within the dermal layer, 40 × . D) Burned hair follicle within the dermal layer with cellular damage including cell swelling, 40 × .

Full-thickness burn was confirmed using histological analysis. Formalin-fixed skin samples were processed using the Leica Histocore Pearl tissue processor, paraffin-embedded using the Histocore Arcadia embedding center, and sectioned using the Leica RM2255 automated rotary microtome. Slides were stained with hematoxylin and eosin (H&E) and examined using light microscopy (Olympus CKX41 Nikon Digital Sight). Compared to non-burned skin, skin samples from animals receiving thermal injury as described showed evidence of injury across all layers, including complete epidermal destruction and thermal coagulative damage to the full thickness of the dermis with involvement of the subcutaneous fat and skeletal muscle (Fig. 4b). Burned hair follicles showed distinct cellular damage and cytoplasmic swelling (Fig. 4d).

The hypermetabolic response associated with severe burns was validated for the thermal injury method. Arterial blood gases and electrolytes (pH, pO_2_, pCO_2_, Cl^−^, Na^+^, K^+^, Ca^2+^, sO_2_, Base Excess, HCO_3_^−^) and lactate were measured using the Radiometer ABL800 blood gas analyser (Radiometer Pacific, Mt Waverley, Victoria), while complete blood counts were obtained using the VetScan HM5 Hematology analyser (REM Systems, Macquarie Park, New South Wales). Lactate and potassium levels were both significantly increased 420 min after burn, while base excess fell from 1.58 to -11.98 mM (p < 0.001) (Table 1). Total white blood cell count increased significantly indicating a systemic immune response (Table 2). While total lymphocytes did not change, there was a significant decrease in the lymphocyte percentage, accompanied by a 4.4-fold increase in neutrophils (p < 0.001). Platelets remained unchanged, however red cells, hemoglobin and hematocrit all significantly increased as plasma leaks from damaged capillaries (Table 2).

**Table 1:** Blood Chemistry Parameters Before and After Burn.

Parameter	Baseline	420min	p-valve
Lactate (mmol/L)	0.67 ± 0.05	1.69 ± 0.23	<0.001
Base Excess (mmol/L)	1.58 ± 1.16	−11.98 ± 1.43	<0.001
Potassium (mmol/L)	4.45 ± 0.23	6.54 ± 0.29	<0.001

**Table 2:** Hematology Parameters Before and After Burn.

Parameter	Baseline	420min	p-value
**White** Blood Cells (10^9^/L)	8.61 ± 1.07	15.16 ± 0.94	<0.001
Lymphocytes (10^9^/L)	6.19 ± 0.74	5.38 ± 0.38	0.340
Monocytes (10^9^/L)	0.41 ± 0.10	0.98 ± 0.14	0.040
Neutrophils (10^9^/L)	2.01 ± 0.38	8.78 ± 0.63	<0.001
Lymphocytes (%)	71.90 ± 2.84	35.75 ± 1.74	<0.001
Monocytes (%)	5.04 ± 1.02	6.30 ± 0.58	0.298
Neutrophils (%)	23.07 ± 2.67	57.94 ± 1.64	<0.001
**Red** Blood Cells (10^12^/L)	6.97 ± 0.23	9.15 ± 0.17	<0.001
Hemoglobin (g/L)	12.81 ± 0.41	16.40 ± 0.29	<0.001
Hematocrit (%)	38.48 ± 1.33	47.92 ± 0.89	<0.001
Platelets (10^9^/L)	221 ± 46	296 ± 19	0.151

### Statistics

SPSS Statistical Package 24 was used for statistical analysis (IBM, St Leonards, New South Wales). All values are expressed as mean ± SEM. Data normality was assessed using Shapiro-Wilk tests and normally distributed data was analysed using an independent-samples t-test. Statistical significance was set as p < 0.05.

## References

[1] Cartotto R., Zhou A. (2010). Fluid creep: the pendulum hasn't swung back yet!. J. Burn Care Res..

[2] Greenhalgh D.G. (2007). Burn resuscitation. J. Burn Care Res..

[3] Blumetti J., Hunt J.L., Arnoldo B.D., Parks J.K., Purdue G.F. (2008). The Parkland formula under fire: is the criticism justified?. J. Burn Care Res..

[4] Rousseau A.F., Massion P.B., Laungani A., Nizet J.L., Damas P., Ledoux D. (2014). Toward targeted early burn care: lessons from a european survey. J. Burn Care Res..

[5] Mitsunaga Junior J.K., Gragnani A., Ramos M.L., Ferreira L.M. (2012). Rat an experimental model for burns: a systematic review. Acta Cir. Bras..

[6] Barber R.C., Maass D.L., White D.J., Horton J.W. (2008). Increasing percent burn is correlated with increasing inflammation in an adult rodent model. Shock (Augusta, GA).

[7] Akscyn R.M., Franklin J.L., Gavrikova T.A., Schwacha M.G., Messina J.L. (2015). A rat model of concurrent combined injuries (polytrauma). Int. J. Clin. Exp. Med..

[8] Huang G., Sun K., Yin S., Jiang B., Chen Y., Gong Y., Chen Y., Yang Z., Chen J., Yuan Z. (2017). Burn injury leads to increase in relative abundance of opportunistic pathogens in the rat gastrointestinal microbiome. Front. Microbiol..

[9] Burger A., Wnent J., Bohn A., Jantzen T., Brenner S., Lefering R., Seewald S., Grasner J.T., Fischer M. (2018). The effect of ambulance response time on survival following out-of-hospital cardiac arrest. Deutsches Arztebl. Int..

[10] Nehme Z., Andrew E., Smith K. (2016). Factors influencing the timeliness of emergency medical service response to time critical emergencies. Prehosp. Emerg. Care.

[11] Rehn M., Davies G., Smith P., Lockey D. (2017). Emergency versus standard response: time efficacy of London’s Air Ambulance rapid response vehicle. Emerg. Med. J.: EMJ.

[12] Wood F.M., Phillips M., Jovic T., Cassidy J.T., Cameron P., Edgar D.W. (2016). Steering committee of the burn registry of a, new z: water first aid is beneficial in humans post-burn: evidence from a Bi-National cohort study. PLoS One.

[13] Ghali M.G.Z. (2017). Microsurgical technique for femoral vascular access in the rat. MethodsX.

[14] Jespersen B., Knupp L., Northcott C.A. (2012). Femoral arterial and venous catheterization for blood sampling, drug administration and conscious blood pressure and heart rate measurements. J. Vis. Exp..

[15] Davenport L., Letson H.L., Dobson G.P. (2017). Immune-inflammatory activation after a single laparotomy in a rat model: effect of adenosine, lidocaine and Mg2+ infusion to dampen the stress response. Innate Immun..

[16] Gouma E., Simos Y., Verginadis I., Lykoudis E., Evangelou A., Karkabounas S. (2012). A simple procedure for estimation of total body surface area and determination of a new value of Meeh’s constant in rats. Lab. Anim..

